# Immunotherapy for liver tumors: present status and future prospects

**DOI:** 10.1186/1423-0127-16-30

**Published:** 2009-03-06

**Authors:** Pablo Matar, Laura Alaniz, Viviana Rozados, Jorge B Aquino, Mariana Malvicini, Catalina Atorrasagasti, Manuel Gidekel, Marcelo Silva, O Graciela Scharovsky, Guillermo Mazzolini

**Affiliations:** 1 Institute of Experimental Genetics, School of Medical Sciences, National University of Rosario, Santa Fe 3100, (2000) Rosario, Argentina; 2 Gene Therapy Laboratory, Liver Unit, School of Medicine, Austral University, Av. Presidente Perón 1500, (B1629ODT) Derqui-Pilar, Buenos Aires, Argentina; 3 CONICET (Consejo Nacional de Investigaciones Científicas y Técnicas), Buenos Aires, Argentina; 4 VentureL@b, Escuela de Negocios, Universidad Adolfo Ibañez, Av. Diagonal Las Torres 2700, Peñalolen 791000, Santiago, Chile

## Abstract

Increasing evidence suggests that immune responses are involved in the control of cancer and that the immune system can be manipulated in different ways to recognize and attack tumors. Progress in immune-based strategies has opened new therapeutic avenues using a number of techniques destined to eliminate malignant cells. In the present review, we overview current knowledge on the importance, successes and difficulties of immunotherapy in liver tumors, including preclinical data available in animal models and information from clinical trials carried out during the lasts years. This review shows that new options for the treatment of advanced liver tumors are urgently needed and that there is a ground for future advances in the field.

## Background

Hepatocellular carcinoma (HCC) is the fifth most common cancer and the third leading cause of cancer-related death worldwide [1]. Unfortunately, the incidence and mortality associated with HCC is increasing steadily [2] as a consequence of epidemics of hepatitis C virus (HCV) and hepatitis B virus (HBV). HCV and HBV infections are causally associated with the majority of HCC in the world [3].

Current therapeutic options are extremely disappointing since less than 30% of the patients evaluated in referral medical institutions can receive a curative therapy, consisting in either resection or transplantation [4]. Thus, in the majority of advanced HCC cases surgery is not possible and the prognosis is dismal due to underlying cirrhosis as well as to poor tumor response to chemotherapeutic agents [4-6].

Unfortunately, advanced colorectal carcinoma (CRC) depict similar scenario [7]. Colorectal carcinoma is one of the most common malignancies and a leading cause of cancer-related death [1]. Hepatic metastases are present in 15–25% of patients at the time of CRC diagnosis [8]. Surgical resection, which is accepted as first-line CRC treatment, cannot be performed in the majority of patients [9]. Following diagnosis, the median survival of untreated patients with liver metastases is 6–12 months [10]. The application of new chemotherapeutic cocktails, including irinotecan or oxaliplatin, result in higher rates of objective responses and survival [11-15] and the recent incorporation of monoclonal antibodies against vascular endothelial growth factor and epidermal growth factor receptors provides additional, although limited, improvement in patients survival [15,16].

Thus, new strategies are needed for treatment of patients with advanced liver tumors and immunotherapy approaches might play a significant role among them. Cancer immunotherapy can be defined as a set of techniques aimed to eliminate malignant tumors through mechanisms involving immune system responses [17,18]. The goal of cancer immunotherapy is to understand how to direct against tumors similar kind of extremely potent immune responses such as those naturally occurring against microbial antigens, and subsequently how to apply these results to human cancer diseases. It has been observed in patients with HCC that the presence of a lymphocyte infiltrate is associated with a better prognosis after resection and transplantation [19]. Similarly, presence of lymphocyte infiltration in tumors was correlated with patient survival in CRC: survival rate of patients with large numbers of CD3+-T cells was 5-years higher [20,21].

**Figure 1:**
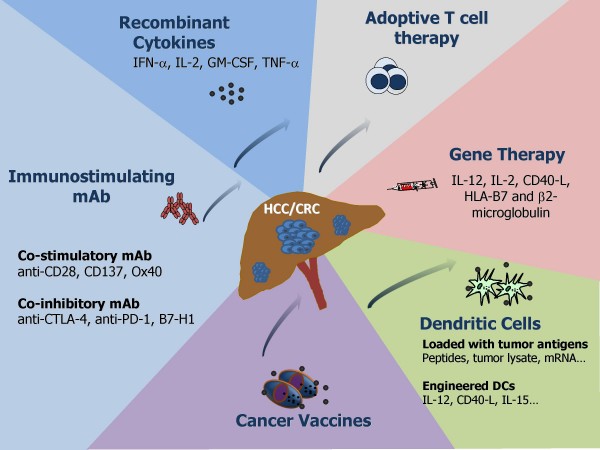
**Immunotherapeutic strategies for liver tumors: administration of recombinant cytokines, adoptive transfer of tumor-reactive T cells generated *in vitro*, gene therapy with cytokines and costimulatory molecules, immunotherapy with dendritic cells, stimulation with immunogenic vaccines or antibodies**.

There is a limited clinical experience regarding the application of immunotherapy in liver tumors contrary to more immunogenic tumors such as melanoma, lymphoma or renal cell carcinoma. Increasing evidence suggests that immune responses are involved in the control of cancer and that the immune system can be manipulated in different ways to recognize and attack tumors (Fig. 1). Unfortunately, the presence of chronic HCV or HBV infection complicates the success of immunotherapy in patients with HCC because these viruses were found to be able to modulate the immune response against tumors and to counteract the immune system of the host [22-24].

## The immune system and the induction of antitumor immunity – basic concepts

The immune system is clearly capable of recognizing and eliminating tumor cells, although cancer cells are considered as poorly immunogenic [25]. Compelling evidence suggests that immune cells can eventually play a crucial role in the control of cancer. First, both occasional spontaneous tumor regressions have been described in immunocompetent hosts while increased cancer incidence has been reported in immunocompromised individuals [26]. Second, tumor immunity was demonstrated experimentally in several animal models [27]. Third, the immune system often recognizes the presence of tumors, as reflected by an accumulation of immune cells at tumor sites [28].

Despite the ability of the immune system to react against cancer cells, the presence of a tumor indicates that the developing cancer can avoid detection or to escape the immune response [29]. Mechanisms used to elude recognition include tumor-induced impairment of antigen presentation, activation of negative co-stimulatory signals, and production of immunosuppressive factors [30]. In addition, cancer cells may promote the expansion and/or recruitment of regulatory cells that may contribute to the immunosuppressive network; these populations include regulatory T cells (Treg), myeloid suppressor cells, and distinct subsets of immature and mature regulatory dendritic cells [31].

All of the previously mentioned mechanisms were shown to be induced in the liver by hepatitis viruses [32,33] and a concomitant chronic HCV/HBV infection in HCC patients would probably make the scenario for immunotherapeutic approaches more complicated.

### The immunosurveillance and the immunoediting hypothesis

In the last 30 years we have witnessed a dramatic change in basic concepts related to tumor immunology, from the strict theory of tumor immunosurveillance postulated by Burnet and Thomas [34,35] to the very recent immunoediting concept developed by Schreiber and colleagues [36]. Using a broader look at tumor immunology, these authors have elegantly described tumor progression as a process following three phases: *elimination*; *equilibrium *and, finally, *escape*, in which tumor cells develop several strategies to avoid their immune-mediated elimination. The variety of processes by which tumors evade the immune response is surprisingly large. Even though cancer cells express new or inappropriate antigens, tumors of diverse origin develop common and/or unique mechanisms that enable them to escape from the immune system.

### The liver: an immunological privileged organ

Mechanisms of tolerance and their implications in cancer are of central interest in immunology. The liver is an especial organ for its immunological privileged status which is a consequence of several unique immunological properties causing antigen tolerance rather than immunity [37,38] and relative resistance against liver allograft rejection [39], allowing that 20% of allotransplanted patients could be withdrawn from long-term immunosuppression [40]. Aggressive autoimmune hepatitis is a somewhat uncommon clinical manifestation of systemic autoimmune disease [41]. Moreover, it has been observed in animal models that naïve liver reactive T cells ignore antigens derived from or expressed in the liver [42], generating tolerance to them [37]. It is important to note that effector T-cells alone may not be sufficient for disease induction without additional inflammatory and costimulatory signals. A potential role for TLR3 has been reported as one of the critical mechanisms of hepatic immune privilege [43].

As it was excellently reviewed by Abe and Thomson [38], liver immunoprivilege properties are likely due to its unique repertoire of antigen-presenting cell (APC) populations, consisting of Kupffer cells (KCs), liver sinusoidal endothelial cells (LSECs) and dendritic cells (DCs). KCs represent 80–90% of liver resident macrophages and are very efficient in clearing LPS from gut-derived blood circulation but less efficient in activating CD4+ cells. LSECs were shown to efficiently separate leukocytes from hepatocytes [44], are able to express factors involved in T cell death, induce differentiation of CD4+ towards the Th2 anti-inflammatory phenotype and were found to co-stimulate Tregs and inhibit allogeneic T cells. DCs are located in portal areas or circulate through liver sinusoids towards lymph draining vessels, and upon maturation increase their expression levels of IL-12 and CCR7, two molecules involved in CD4+ T cell differentiation towards the Th1 pro-inflammatory phenotype and in DC trafficking towards secondary lymphoid organs, respectively. From all liver APCs, DCs are the most potent to elicit immune responses. Due to the fact that KCs and LSECs constitutively express IL-10 and TGF-beta anti-inflammatory cytokines, T cell differentiation is affected and APC maturation inhibited in the liver [45,46]. As a consequence, the DCs are less immunostimulatory than in spleen [47,48].

In addition, hepatic stellate cells (also known as Ito cells) were shown to be involved in liver immunological processes only in case of chronic liver injury. They are induced to transdifferentiate into myofibroblasts and to secrete a number of cytokines and chemokines, such as transforming growth factor beta (TGF-beta) [49,50]. In fact, activated hepatic stellate cells have been shown to closely interact with lymphocytes [51] and to have potent antigen-presenting properties [52]. Furthermore, stellate cells from hepatitis patients have been shown to get further activated by lymphocyte proximity, especially by CD8+ cells, and to phagocyte CD45+ cells [53]. Those facts suggest that stellate cells are likely implicated in the down-regulation of the immune response in HCV/HBV-derived cirrhosis and might also be involved in HCC. These findings open new therapeutic opportunities aimed to specifically targeting hepatic stellate cells in advanced cirrhosis and HCC.

Finally, when HCC coexists with HBV/HCV derived cirrhosis, these viruses as shown in chronic hepatitis, would likely exert direct and indirect effects on further downregulation of the immune response through complex and not fully understood mechanisms. They might influence the activity of hepatic stellate cells as well as that of resident and recruited immune cells, such us DCs, through direct viral protein interaction [54-57]. As reviewed by Liu et al. [33] in chronic B/C-viral hepatitis a reduction in the myeloid and plasmacytoid DC liver populations, down-regulation in IL-12 and IFN-gamma levels, an up-regulation of IL-10 and an impairment in DCs capacity to prime naïve T cells may account for the insufficient immune response observed. Similarly, a reduction in circulating DC numbers was found in the peripheral blood of patients with either chronic-B-hepatitis [58] or chronic-C-hepatitis [59,60]. HBV/HCV viruses would likely contribute to the DC impaired allostimulatory and IL-12 production capacities observed in HCC patients [61], although this remains to be elucidated.

### Hepatic tumors escape from the immune response

Hepatic tumors use two main strategies to escape from the immune response – **attack **and **defense **– the first is designed to **attack **the immune cells, hence avoiding their antitumor action and the other to **defend **tumor cells by enabling them to pass unnoticed by the immune response (Table 1).

**Table 1:** Mechanisms of hepatic tumor-immune escape.

**Attack**			**Defense**		
**System**	**Mechanism**	**Ref**.	**System**	**Mechanism**	**Ref**.

Fas/FasL	T-cell apoptosis	66	Tregs	Immunosuppression	73

PD-1/PD-1L	T-cell apoptosis	69	MHC-I	Antigen presentation	75

Galectin-1	T-cell apoptosis	72	B7-1/B7-2	Antigen presentation	76
			
			IDO	Immunosuppression	82

Two main strategies to escape from the immune response attack and defense have been demonstrated for HCC in experimental and/or clinical setting. Fas: CD95; FasL: CD95L; PD-1: programmed death-1; PD-1L: programmed death-1 ligand; Tregs: Regulatory T cells; IDO: Indoleamine 2,3 dioxygenase.

#### Attack strategies

Fas ligand (FasL), a type II transmembrane protein reported to induce apoptosis of Fas-bearing cells [62] was shown to confer immunological privilege to certain tissues and organs such as eye, placenta and central nervous system [63-65]. More recently, the interaction of FasL or its secreted isoform (sFASL) produced by tumor cells, with their specific Fas receptor, expressed on T lymphocytes, was implicated in tumor cell evasion from immune surveillance [66]. The α-fetoprotein (AFP), an oncofetal protein overexpressed in some HCC, was shown to induce Fas-L and tumor necrosis factor [TNF]-related apoptosis expression in HCC Bel7402 cells, as well as TRAIL receptor and Fas in lymphocytes [67,68]. Another pathway developed to attack immune cells involves the interaction of PD-1 (programmed death-1) with its ligands PD-L1 and PD-L2. Immunotherapy with an expression plasmid encoding the extracellular domain of PD-1 (sPD-1) in H22 HCC cells was shown to improve the immune response against tumors [69]. One further mechanism might implicate Galectin-1 (Gal-1) – a β-galactoside binding protein with immunoregulatory properties, which is known to play a role in cytotoxic immune cells elimination. It is likely that Gal-1 contributes to tumor immune escape by killing activated T cells [70,71]. In fact, the expression of Gal-1 was shown to be induced in primary HLF, HuH7 and HepG2 cells [72].

#### Defense strategies

The pressure that the immune system exerts on the growth of tumor cells seems to have led them to develop several protection mechanisms against any immune attack. It has been shown that human HCC-related factors not only induce and expand the regulatory CD4+CD25+ T cell population (Tregs), but also enhance their suppressor ability [73]. A high prevalence of Tregs infiltrating HCC seems to be an unfavorable prognostic indicator [74]. Another mechanism frequently used by tumors is the down-regulation of MHC-I [75], B7-1/B7-2 co-stimulatory molecules [76] or transporter associated with antigen processing (TAP)1/2 molecules in human HCC [77]. In addition, HCC cells might escape from CTL-induced apoptosis by increasing Bcl-2 and decreasing Bcl-xs expression [78] and/or raising the Survivin level, an important member of the inhibitor of apoptosis (IAP) family [79,80].

Indoleamine 2,3 dioxygenase (IDO) catalyses the degradation of the essential amino acid tryptophan and synthesizes immunosuppressive metabolites [81]. Larrea and colleagues [82] reported that IDO constitutes an important mediator of peripheral immune tolerance in chronic hepatitis C virus (HCV) infection. Induction of IDO expression may reduce T-cell reactivity to viral antigens in chronic HCV infection and may also influence the immune response against HCC in patients chronically infected with HCV. Understanding of the immune-escape mechanisms should help us to design immunotherapy protocols to increase the efficacy of therapeutic success.

## Systemic use of immunostimulatory cytokines

There is a broad experience regarding the use of cytokines to induce immune and inflammatory responses against cancer [83,84]. Cytokines have been shown to act through different mechanisms: i) stimulation of antitumor immune responses; ii) induction of tumor cell apoptosis (e.g. through induction of TRAIL) [85]; iii) interference in uncontrolled proliferation of cancer cells, and iv) anti-angiogenesis.

One of the most explored cytokines is interferon alpha (IFN-α) [86,87]. The IFN-α antitumor mechanism of action includes direct effect on tumor cells, induction of lymphocyte and macrophage cytotoxic activities and anti-angiogenesis [88,89]. Two controlled trials comparing IFN-α with symptomatic treatment in patients with HCC were reported. In one of them the use of high doses of IFN-α (50 MU/m2, tiw) resulted in a response rate of 36% [90]. In the other trial, in which lower doses of IFN-α (3 MU/m2, tiw) were administered, the response rate was poor (7%) [91]. Even though it is clear that the different responses are related to the administered doses, the toxicity associated with the higher IFN-α dose is not acceptable, especially for patients with end-stage liver disease. Nevertheless, systemic administration of IFN-α [92] or IFN-β [93] should be considered as a supportive treatment after hepatectomy or tumor ablation, which may prevent or delay tumor relapses in patients with HCC [94]. A combination of IFN-α and chemotherapy was applied to patients for treatment of advanced HCC [95,96] and metastatic CRC to the liver [97]; however, randomized controlled studies failed to demonstrate that combination protocol results in improved outcome when compared to chemotherapy treatment alone [98,99].

Interleukin-2, an immunostimulatory cytokine, has been administered alone or in combination with other treatments against liver tumors. The non-controlled nature of most studies precludes from any definitive conclusion. Systemic IL-2 was able to produce objective responses against HCC when given alone [100] or in combination with melatonin [101] or lymphokine activated killer (LAK) cells [102]. On the other hand, hepatic artery infusion of interleukin 2, with or without chemotherapy, induced objective remissions in 5% to 15% of liver metastases from CRC [95,103,104]. In a phase II clinical trial, Correale and colleagues showed that the combination of polychemotherapy with granulocyte macrophage colony-stimulating (GM-CSF) factor and low-dose IL-2 in colorectal carcinoma patients, results in high number of objective responses and low toxicity [105].

There is one report on combination of hepatic trans-arterial chemotherapy with IFNγ plus IL-2 in patients with advanced HCC [106]. The achieved objective responses highlight some biological effect of this treatment combination. In another study, when IL-2 was administered together with IFNγ and GM-CSF to advanced HCC patients, clinical results were poor [107]. However, in spite of some stimulating results, the clinical development of IL-2 has been proved unsuitable because in parallel to their efficacy the results involved severe toxicity, including systemic vascular leak syndrome.

No trials were reported on the application of other cytokines such as IL-12, TNFα, or TRAIL, known to have a potential effect against primary or metastatic liver cancer in humans. Nevertheless, concerns were raised following reports on the development of severe toxicity after systemic treatment with IL-12 or TNFα [108,109] in other type of tumors.

Although being able to obtain some positive outcomes in the treatment of liver tumors, systemic application of cytokines is accompanied by toxic effects which can be overcome by local delivery. A possible role of some of the immunostimulatory cytokines, e.g. IL-12, could be reasonable in the context of vaccination as an adjuvant administered at low doses.

## Immunostimulating monoclonal antibodies

In the field of cancer therapy mAbs can act directly against tumor cells or indirectly by interfering with several processes such as survival, cellular proliferation or angiogenesis. The immunostimulating monoclonal antibodies which are those corresponding to the latter group, are defined as a new family of drugs aimed to augment immune responses. They consist in either agonistic or antagonistic mAbs which are aimed to bind key immune system receptors, thereby enhancing antigen presentation, providing co-stimulation or counteracting immune-regulation [110].

### Regulation of T-cell responses

T-cells express several co-signalling molecules, typically cell-surface glycoproteins classified as co-stimulators or co-inhibitors [111,112]. The outcome of T-cell responses depend on the balance between co-stimulatory and co-inhibitory molecules. Thus, antigenic signalling in the absence of co-stimulatory molecules results in suboptimal immune activation and may lead to T-cell deletion or unresponsiveness. Monoclonal antibodies targeting co-stimulatory molecules expressed on T-cells may act agonistically, working as surrogate ligands and augmenting T-cell proliferation and survival. Alternatively, mAbs may act antagonistically, counteracting the inhibitory effects of co-inhibitor molecules or Treg-cells.

### Costimulation with agonistic mAbs

Diverse costimulatory molecules appear to regulate T-cell response, working specifically at different time points [113,114]. Antibodies against CD28 are known to potentiate antitumor immunity in combination with bi-specific antibodies that bind to both the tumor antigen and the TCR-CD3 complex [112]. Some anti-CD28 antibodies, termed *superagonist *antibodies, can activate T-cells without concomitant TCR engagement. Unfortunately, concerns were raised following reports of severe toxicity in a Phase I dose-escalation trial with an anti-CD28 mAb (TGN1412) [115].

Another costimulatory molecule, CD137 (also known as 4-1BB), is a member of the TNF-receptor superfamily, expressed in antigen-activated T-cells (CD4+, CD8+, Treg and NK cells), DCs, cytokine-activated NK cells, eosinophils, mast cells and, intriguingly, endothelial cells of some metastatic tumors [116-118]. The natural ligand for CD137 (CD137 ligand) is constitutively produced by activated APCs. Agonistic anti-CD137 Abs strongly promote survival of T-cells and prevent activation-induced cell death [119,120]. Antitumor effects of anti-CD137 mAbs were first recognized by Melero et al. [121] in established Ag104 sarcoma and P815 mastocytoma. These effects are thought to be involved in the activation of naive T-cells which are specific for tumor antigens cross-presented by DCs. Repeated systemic injections of agonistic anti-CD137, in two mouse models of CRC, induced tumor eradication in 3 out of 5 mice [122]. Unfortunately, this therapeutic modality may have serious drawbacks. Niu and colleagues found that a single injection of anti-CD137 given to BALB/c or C57BL/6 control mice led to the development of a series of anomalies such as splenomegaly, lymphadenopathy, hepatomegaly, multifocal hepatitis, anemia, altered trafficking of B cells and CD8+ T-cells, loss of NK cells, and a 10-fold increase in bone marrow cells bearing the phenotype of hematopoietic stem cells [123].

OX40 (also known as CD134 and TNR4) is another member of the TNF receptor family, specifically expressed in activated CD4+ and CD8+ T lymphocyte, B-cells, DCs and eosinophils [124]. OX40 ligand (OX40L) is expressed in activated APCs and can also be found in activated T-cells and in endothelial cells [125]. OX40 seems to be particularly important to ensure T-cell long-term survival, probably through up-regulation of the anti-apoptotic proteins Bcl-xL and Bcl-2 [126]. Weinberg [127] showed that systemic OX40 ligation increases tumor immunity, with a role for CD4+ cells in the B16 melanoma model. Phase I clinical trials, using a murine anti-human OX40 mAb, have been initiated in patients with advanced cancer of multiple tissue origins; however, it can not be administered in several repeated doses because of its xenogeneic nature, which is likely to trigger immune responses against murine sequences [128].

Thus, agonistic mAbs have been found to produce some benefits in treatment of liver tumors although their systemic application causes serious undesired secondary effects. Intratumoral application of low doses of them might overcome some of the systemic delivery problems.

### Counteracting immunoregulation with antagonistic mAbs

The cytotoxic T-lymphocyte-associated protein 4 (CTLA-4, also known as CD152) is an inhibitory receptor with a structural homology to the co-stimulatory receptor CD28 [111,129]. Under antigenic stimulation, ligand binding to CTLA-4 generates inhibitory signals mediating reduction in T-cell proliferation and in IL-2 secretion. Administration of antagonistic anti-CTLA-4 mAbs demonstrated antitumor effects in different murine tumor models including colon, prostate and renal carcinomas, as well as fibrosarcoma and lymphoma [130,131].

As mentioned earlier, PD-1 and its ligands B7-H1 (also known as PD-L1) and B7-DC (also known as PD-L2) [111,132] deliver inhibitory signals to T cells. Administration of mAbs anti-PD-1 and B7-H1 produced CTL-mediated antitumor effects in mice [133].

The finding that HCC-associated antigen HAb18G/CD147, a member of the CD147 family, enhances tumor invasion and metastasis through induction of matrix metalloproteinases [134] led to the development of an anti-CD147 therapy. By using an orthotopic model of HCC in nude mice, Ku and colleagues [135] showed that the application of two different anti-CD147 mAbs (HAb18 and LICARTIN) resulted in consistent inhibition of both tumor and metastasis growth.

In animal models, immunostimulatory mAbs antitumor effects were demonstrated when used either alone or in combination with radiotherapy or chemotherapy [136,137]. Clinical experience with mAbs is scarce; however, several immunostimulatory mAbs have now been introduced in clinical trials and early results suggest that they might enhance antitumor responses with accepted toxicity. Therapy with immunostimulatory antibodies alone or in combination with other strategies should be carefully designed in order to avoid induction of autoimmune toxicity as a consequence of uncontrolled stimulation of the immune system effector arm.

## Gene transfer of cytokines and costimulatory molecules. Genetic vaccination

Gene therapy is a promising novel therapeutic strategy for treatment of several heritable and non-heritable human diseases [138,139]. Since about 20 years ago, when the first clinical trial was initiated, and after more than 1300 clinical trials performed all around the world , we learned that the core concept of gene therapy may be applicable: genes introduced into patients can be safely expressed [140]. However, we have also learned that vector efficiency in clinical applications is not as good as expected [141,142]. Cancer represents almost 70% of the clinical trials conducted in patients and 25% of these studies consisted in the application of cytokine genes.

Gene transfer of immunostimulatory cytokines (e.g. IL-2, IL-4, IL-6, IL-7; IL-12, INF-γ, TNF-α, GM-CSF) was shown to overcome the immune tolerance against tumors, facilitating their eradication in some cases [143-145] (Table 2). Two main approaches have been used [144]: i) direct injection of vectors expressing cytokines/chemokines/costimulatory molecules into tumor lesions, or ii) use of tumor cells/DCs transduced *ex vivo *with vectors expressing cytokines/costimulatory molecules.

**Table 2:** Gene transfer immunostimulatory molecules.

**Cytokine**	**Mechanism**	**Ref**.
IL-2 + IL-12	CTLs	149

IL-10	CD8+	152

IL-12	NK, CD4+, CD8+	187

IL-12 + IL-10	NK, CD4+, CD8+, Macrophages, Neutrophils	165

IL-2	CTLs	166

HLA-B7	CTLs	167

IL-12 + IP-10	CD8+, CD4+, NK	161

IL-12 + MIP3α	CD8+, CD4+, NK	162

CD40-L	CD8+	168,169

GM-CSF + HSV	CD4+, CD8+	171

CTLs: Cytotoxic T lymphocytes; NK: Natural killer cells.

Interleukin 12 (IL-12) is a potent cytokine that showed antitumor activity in a number of tumor models [146,147]. Multiple action mechanisms mediating its activity are known, including the activation of NK cells, cytotoxic T lymphocytes and the induction of a TH1 type of response [146]. It also inhibits tumor angiogenesis and enhances the expression of adhesion molecules on endothelial cells, thus facilitating the homing of activated lymphocytes to the tumor [148,149]. However, IL-12 was shown to eventually induce severe toxicity when administered systemically as a recombinant protein [150]. Thus, unspecific toxic effects of systemic IL-12 administration might be solved by the use of gene therapy strategies, allowing local production of IL-12 at the tumor milieu and resulting in high local levels with low systemic concentrations [151]. Consistently, the potential usefulness of IL-12 gene transfer for liver tumors treatment in animal models was demonstrated by different groups including ours [152-154]. We also reported that intratumor injection of an adenovirus encoding IL-12 genes (AdIL-12) into rats with orthotopic HCC induced the complete tumor elimination in the majority of animals [155]. Potent effects of this vector have also been shown in a very aggressive multifocal HCC model developed in rats, by treatment with DENA [155,156] as well as in mice bearing hepatic metastases of colorectal carcinoma [157,158] and in woodchucks chronically infected with woodchuck hepatitis virus (WHV) [159]. The toxicity observed under high IL-12 levels is partly due to induction of IFN-γ overproduction [160]. An encouraging result is that IL-12 gene transfer in combination with another vector expressing the chemokine IP-10 (AdIP-10) allowed the reduction in the AdIL-12 dose with a similar outcome efficacy [161]. The underlying mechanism is the following: lymphocytes get attracted to tumors due to a local IP-10 expression and subsequently they are activated by IL-12. In addition, a combination of IL-12 with MIP3α demonstrated similar synergistic antitumor effects [162].

The effects of IL-12 gene transfer were assessed in patients with advanced gastrointestinal carcinomas in a phase I clinical trial consisting mainly in liver tumors. Patients were administered with up to 3 intratumor injections of AdhIL-12[163]. Treatment feasibility and safety were studied. Even though maximal tolerated dose has not been reached, some evidence of biological and antitumor activities were observed. One partial response, two minor regressions and six stabilizations were achieved. In four out of 10 patients, a significant lymphocyte infiltrate was observed in injected tumors.

It has been stated that abnormal elevated levels of Th2 cytokines such as IL-10 are able to skew an immune response that favors tumor growth [164]. In contrast, Lopez et al. [165] have recently shown that tumor cell vaccines producing a combination of IL-10 and IL-12 act synergistically in eradicating established CRC, with the underlying mechanisms being not fully addressed.

Systemic injection of recombinant IL-2 used extensively in clinical oncology for patients with metastatic renal carcinoma and melanoma has shown low efficacy and high toxicity. A phase I-II clinical trial consisting in the administration of a recombinant adenovirus encoding for IL-2 gene was carried out in patients with advanced digestive carcinomas [166]. Only one of the treated patients showed a positive tumor response with necrosis of the tumor mass.

Molecules such as HLA-B7 are essential to promote specific T-cell responses. A reduced expression of MHC-I was observed in CRC. In an attempt to make CRC more visible to the immune system, Rubin et al [167] carried out a phase I clinical trial consisting in an indirect intralesional gene transfer of both HLA-B7 and β2-microglobulin into CRC hepatic metastases. Treatment with a single plasmid construction encoding for both genes in a lipid formulation (Allovectin-7) was feasible and safe in 15 patients, however, details regarding antitumor effect have not been reported. Such an approach could produce significant therapeutic improvements if aimed to deliver functionally relevant genes.

The interaction between CD40 ligand (CD40L, CD154) and its receptor CD40, expressed in DCs, is essential for the initiation of cellular and humoral immune responses. Gene transfer of CD40-L led to regression of established CRC [168] and HCC [169] in a CD8+ T cell dependent manner.

Replication-selective viral agents (oncolytic virotherapy) hold promise as a novel cancer treatment platform. Oncolytic virotherapy is based on the ability of these vectors to selectively replicate in cancer cells as a result of different mechanisms of action [170]. This novel class of targeting viral vectors exerts direct antitumor effects, but can also be engineered to produce immunostimulatory genes, such as GM-CSF, augmenting its efficacy. A potent *in vivo *antitumor effect of an oncolytic vector carrying HSV and GM-CSF genes has been demonstrated against murine CRC CT26 and murine HCC Hepa 1.6 [171].

The mutant adenovirus dl1520, also called ONYX-015, was the first described oncolytic adenovirus [172]. It contains a deletion in the E1B 55 K gene that achieves preferential replication in cancer cells by different mechanisms. In the case of liver tumors, this virus showed a partial antitumor effect on murine models but no evident antitumor effect was found when applied to HCC patients. Two separate clinical trials showed that ONYX-015 has limited therapeutic effect as monotherapy in patients with liver tumors, especially if systemic routes are used [173,174]. Other oncolytic adenoviruses have been developed, and show promising results in animal models of HCC. However, their performances in clinical trials have not been tested so far [175].

In conclusion, gene transfer of cytokines and the use of oncolytic viruses are two developing immunotherapy strategies which hold promise in treatment of liver tumors. The former strategy is being widely applied and after further improvements might assure sufficient tumor levels of inflammatory cytokines circumventing toxic systemic effects. The latter strategy is in early stages of development and it largely needs to be applied into the clinics.

### Immunotherapy with dendritic cells

The armamentarium for immunotherapy protocols has been boosted by the identification of DCs as protagonists of antigen presentation [176]. The final outcome of DC cross-presentation could be either T-cell activation or T-cell tolerance, depending on its activation/maturation status [177]. Thus, while mature DCs are able to induce antitumor immunity, antigen presentation by immature DCs results in the induction of tolerance [177]. In addition, IL-4 which is overexpressed in the liver under recurrent hepatitis C [178] was shown to influence DCs to induce CD4+ T cell differentiation into the Th2 lineage and to suppress DC response to IFN-gamma [179]. Up to now, several clinical studies consisting in the application of DCs were performed and, as a general outcome, no significant side effects were observed in the majority of these trials with important biological effects showing the augmentation of cellular immune responses against tumor antigens [180].

Direct injection of DCs into tumor tissue has been exploited experimentally and clinically with diverse results [181-183]. Chi KH and colleagues [184] conducted a phase I trial in patients with advanced HCC after conformal radiotherapy. Intratumoral injections of autologous immature naïve DCs prior and after radiotherapy resulted in 2 partial and 4 minor responses. Induction of specific immune responses against AFPs and enhancement in NK activity were observed.

DCs *ex vivo*-engineered to produce IL-12 were shown to induce antitumor immunity in mice [182,183]. Similar results were reported after application of DCs genetically modified to express IL-7 [185] or IL-15 [186]. A phase I clinical trial consisting in the intratumoral injection of autologous DCs, transfected with Ad-IL-12, in patients with metastatic gastrointestinal carcinomas was carried out [187]. This strategy was feasible and very well tolerated in doses up to 50 × 10^6 ^DCs. One partial response and 2 stabilizations were observed. In 3 out of 10 treated patients, a marked increased in CD8+ T lymphocyte infiltrates was found, and in 5 of them NK activity was significantly induced. One of the possible reasons behind the limited antitumor activity might be that DCs would likely be retained within the malignant tissue due to increased intratumoral levels of IL-8 expression as well as other chemotaxis signals, preventing their mobilization to the secondary lymphoid organs for further amplification of immune responses. Consistently, scintigraphic tracking of injected ^111^In-labelled DCs showed retention of DCs inside tumors [188].

As previously discussed, CD40-L is a costimulatory molecule expressed mainly on activated CD4+ T cells, which is essential for the initiation of antigen-specific T-cell responses [189]. Crystal and colleagues [190,191] showed elimination of CRC nodules after intratumoral administration of CD40-L exogenously expressing DCs. Although this approach has not yet been applied in clinical trials, it seems promising.

Another technique employed to load antigens to DCs consists in the cellular transfection with mRNA molecules. Chu et al. transfected total mRNA from CT26 CRC cells to DCs and showed strong specific CTL activity as well as protective immunity *in vivo *[192]. Immunization of CEA-transgenic mice, using mature DCs loaded with an anti-idiotype antibody that mimics CEA, resulted in a potent antitumor response against CEA-expressing CRCs, while immunization with DCs loaded with CEA showed less potent response [193]. Morse et al. reported a phase I clinical trial consisting in the administration of autologous DCs loaded with CEA RNA (peptide CAP-1) into 21 patients with resected CRC liver metastases [194]. The procedure was well tolerated, one patient had a minor response, and one had stable disease. More recently, the same group carried out another phase I study in 14 patients (12 CRC and 2 non-small lung cancer) on the effects of immunotherapy combined with DCs transduced with a fowlpox vector encoding CEA and costimulatory molecules. Immunization of these patients was safe and it was able to activate potent CEA-specific immune responses. In a phase I clinical trial with the aim of increasing the amount of circulating DCs, Fong et al. incubated DCs with the hematopoietic growth factor Flt3 ligand before injecting DCs loaded with CEA-derived peptide into 12 patients with colon or non-small cell lung cancer [195]. Two patients showed objective responses and two had stable disease.

Stift and colleagues reported that vaccinations with autologous DCs pulsed with tumor lysates in a cohort of advanced cancer patients (including two with HCC) was safe and feasible [196]. Delayed-type hypersensitivity (DTH) skin test was positive in the majority of vaccination-treated patients and induction of IFN-γ producing T cells was achieved in 4 other patients (not HCC). Another similar DC-based strategy was applied by Iwashita and colleagues [197]. They carried out a phase I clinical trial in patients with advanced HCC. DC-based strategy consisted in the subcutaneous injection of DC pulsed with tumor extract in 10 patients. One patient showed a partial response and in 2 of them AFP levels were decreased. Seven out of 10 showed positive DTH tests for KLH. Tamir and colleagues [198] evaluated the effectiveness of tumor-lysate loaded DC vaccines in the treatment of advanced CEA-positive CRCs.

Itoh et al. combined both DCs pulsed with a CEA peptide (restricted to HLA-A24) and adjuvant cytokines (IFN-α and TNF-α) in the treatment of patients with CEA-expressing metastatic tumors [199]. Ten HLA-A24 patients with advanced digestive tract or lung cancer were treated. No significant adverse effects were observed and the disease in 2 positive DTH test was stabilized [200]. A few years later, Ueda and colleagues conducted a phase I clinical study in which DCs previously pulsed with a CEA-derived peptide were administered to HLA-A24-restricted patients. Eighteen compatible patients were enrolled. No severe toxicity was observed. In some patients, stabilization of the disease and decrease in CEA levels were reported. Accordingly, patients with clinical responses were positive in skin tests and developed specific CTLs [201]. Finally, Babatz and colleagues demonstrated that immunotherapy with DCs pulsed with a CEA-derived peptide is able to induce specific IFN-gamma producing CD8+ T cells [202].

We and others have observed that DCs and NK cell interaction plays an important role in tumor immunity [187,203,204]. In this regard, Osada and colleagues found in patients with metastatic CRC that immunization with DCs transduced with a fowlpox vector encoding CEA was able to increase NK activity in 4 of 9 patients [205]. Importantly, increased NK activity was correlated with clinical response. In order to *in vivo*-activate DCs and thereby avoiding *ex vivo *manipulation, Furumoto et al. injected MIP3α chemokine together with CpGs inside CRC tumors [206]. They observed an increase in DC number within tumors which were finally eradicated through the development of specific CTLs.

The use of cytokines as a vaccine adjuvant has been shown to be a promising option for cancer therapy, due to its potential effectiveness against disseminated disease without causing systemic toxicity [207-211]. However, the weakness of these strategies lies in: 1) the need of culturing autologous cancer cells from each patient, 2) the problems in the selection of positively modified cancer cells, 3) the lack of an efficient APC activity in tumor cells and, 4) the limited amount of tumor cells that precludes repeated immunizations. Investigators have looked into other strategies to carry cytokines genes or tumor antigens (such as the use of allogeneic tumor cell lines) but, unfortunately, allogeneic tumor cells may lack sole TAA present within the patient's own tumor, thus reducing its efficacy.

In conclusion, different strategies involving DCs have been developed during the lasts years. Although for some of them no clinical trials have been conducted yet, for other strategies a proportion of patients responded to treatment with minor tumor regression or stabilization, with variable induction of the immune response. Further studies are required for improving the benefits of manipulating the main kind of APCs involved in immune reactions.

## Contribution of adoptive T-cell therapy strategies

In several animal models, solid tumors were shown to be susceptible to elimination after infusion of large amounts of tumor-specific T-lymphocytes [212]. However, the translation of these enthusiastic successes into patients are not yet feasible, partly due to difficulties in generating tumor antigen-specific T-cells *ex vivo *[213].

Adoptive therapy involves the transfer of *ex vivo *expanded and stimulated immune effector cells to tumor-bearing hosts, aiming at augmenting the antitumor immune response [212,214]. In general, adoptive therapy is accomplished by harvesting cells from the peripheral blood, tumor sites (tumor infiltrating lymphocytes), or draining lymph nodes from which, the effector cells could eventually be expanded *ex vivo*, in either a specific or non-specific fashion.

One of the major aims of the adoptive T-cell therapy is the identification of tumor-associated antigens (TAAs) that are ectopically expressed or overexpressed in tumor cells relative to normal tissues or, tumor-specific antigens (TSAs) that are expressed exclusively in tumor cells. Despite aberrant expression of TAAs in tumor cells, many of these proteins are also expressed at some level in non-malignant adult tissues and, as a consequence, the immune system may recognize TAAs as self-antigens and limit the T-cell immune response. In addition, as previously discussed the liver immune system usually generates tolerance to proteins expressed by its own cells and HCC induces immune response suppression [215]. Moreover, it was demonstrated that many malignant tumors find the way of down-regulating, modifying or losing its own antigens, in order to avoid immune recognition [29].

No TSA with high prevalence have been identified for liver tumors, so far. PLAC-1, which in normal tissues is only expressed in placenta, was recently found to be expressed in 1/3^rd ^to 1/4^th ^of the analyzed human HCC samples and 3,8% of patients were shown to present humoral responses against this antigen [216]. Among TAAs described in HCC the most important one is AFP. Several AFP-based immunotherapeutic approaches have been applied against HCC [217,218]. Additional TAAs recently found to be expressed in HCC are several members of the tumor-specific "cancer-testis" antigens (the MAGE, GAGE and BAGE genes, NY-ESO, CTA, TSPY and FATE/BJ-HCC-2, among others) [219-221];, Aurora-A [222], SCCA [223], and Glypican-3. In between them, Glypican-3, a specific immunomarker for HCC that can be used to distinguish it from benign hepatocellular mass lesions, is highly immunogenic in mice and can induce effective antitumor immunity with no evidence of autoimmunity [224]. Several TAA antigens are also known for CRC liver tumors, including CEA and CP1 [225]. Clinical studies must be conducted in order to evaluate the potential use of these antigens in immunotherapy for liver tumors.

The lack of TSAs for HCC may be the most important limit to immunotherapy applications aimed to specifically target liver tumor cells. Several technological strategies such as serologic recombinant expression cloning (SEREX), gene expression profiling and proteomics, are being applied to discover any of those specific markers [226] but, until now, the results are limited [227].

Another important negative factor limiting the success of this type of immunotherapy is the low survival of adoptively transferred T-lymphocytes in cancer patients is. Currently, some strategies are being evaluated to increase the proliferation rate of transferred T-cells, including pre-treatment with cyclophosphamide [228].

T cells are the cellular model predominantly chosen for adoptive cellular therapy, although a role for NK cells and other cytokine-induced lymphocytes have also been investigated. Pilot clinical trials of adoptive T cell immunotherapy were initiated in cancer soon after the discovery of IL-2 (in the late 1970s), which enabled large-scale culture of T cells [229]. Although certain clinical success has been observed in melanoma, renal cancer, and lymphoma [230,231], phase II studies in HCC patients have shown objective response rates of only about 20% [232,233].

To date, no randomized clinical trials, but one, had demonstrated efficacy of adoptive T cell transfer approaches. Takayama et al. [234] reported benefits of adoptive transfer with an adjuvant setting for HCC after surgical resection of the primary tumor. In this study, autologous peripheral blood T cells were pre-cultured in medium supplemented with CD3-specific antibody and IL-2, and cell infusion was shown to reduce the risk of cancer recurrence by 41% when compared to a control group receiving only surgery. However, this trial remains unconfirmed, and the mechanism involved in the antitumoral effect remains unknown.

In order to enhance the effector capacity of tumor-specific T cells, different cytokines such as IL-18 and IL-12, were tested as potential biological response modifiers in the setting of adoptive immunotherapy. Nakamori et al. [235] demonstrated that adoptive transfer of IL-18-transduced cytotoxic T-lymphocytes in combination with IL-12 showed marked inhibitory effects on primary tumors and metastasis in a mouse model of orthotopic CRC.

## Synergistic effect of combined therapy

Combinatorial strategies against cancer could either consist in a simultaneous application of different immunotherapeutic approaches or in a combination of classic chemo- or radio-therapeutic protocols with immunologic tools. Some chemotherapeutical agents were shown to induce upregulation of tumor-associated antigen expression (such as CEA) or to reduce tumor cell resistance to specific cytotoxic T lymphocytes. Some of these combinations have been found to produce synergistic rather than additive effects.

The immune-inhibitory mechanisms developed by tumor cells, such as overproduction of immunosuppressive cytokines (TGF-β and IL-10) or induction of Treg cells, are important obstacles that a successful cancer immunotherapy strategy has to face. Inhibition of one or more of these mechanisms appear to be a good strategy to induce antitumor immunity [236]. Elimination or inhibition of Treg activity by low-dose cyclophosphamide [237] or antibodies against CD25 or CTLA-4 may modify tumor immunosuppressive microenvironment, thereby increasing the efficacy of immunotherapy.

It has been shown, both in mice and humans, that pre-treatment with cyclophosphamide, known to induce lymphodepletion, results in a sustained function of adoptively transferred T-cells. Adoptive transfer efficacy can also be enhanced by alternative immunotherapies such as cytokine administration [238] and in some cases by standard cytotoxic chemotherapy and radiotherapy [239,240].

Preclinical models support the rationale for combining cancer vaccines with conventional therapies, such as radiation, chemotherapy, surgery, hormone therapy, as well as other immunotherapies. One of the most promising results was obtained from clinical trials combining antibodies against CTLA-4 with other immunotherapies such as application of GM-CSF-transduced tumor-cell vaccines. This treatment resulted in the alteration of the intratumor balance of Tregs-T effector cells and in tumor rejection [241]. Further research is required to optimize the combination of different immunotherapies to obtain maximal clinical benefits.

## What have we learned from the clinic? Conclusion

Conducting immunotherapy clinical trials in patients with liver tumors is challenging and several strategies have been opened for clinical applications. However, the high efficacy of different immunotherapy strategies at eliminating liver tumors in animal models is in contrast with the very limited results achieved in patients. There are many explanations to why immunotherapy strategies fail or have little impact on patient survival. In general, for all solid tumors, the common scenario chosen to test immunotherapeutic protocols almost always involves patients with advanced diseases that precludes, or at least decreases, the possibility of success. Then, due to the advanced status of the cancer disease, the immune system of the majority of treated patients is deteriorated and unable to recognize tumor antigens. For the specific case of HCC and partially to CRC liver tumors, apart from the immunological privilege status of the liver, there are some particular aspects that add further difficulties when aiming for a clinical response such as the immunosuppressant effect of chronic HBV/HCV infection on cells of the immune system (e.g. DCs) or complications derived from developed cirrhosis which usually undermine efforts to stimulate the immune response. There is a general agreement in that different forms of immunotherapy should be tested for overall clinical benefits along with conventional treatment regimens evidencing improvements in survival. It would be desirable to evaluate the possibility of immunotherapy strategies as neoadjuvancy in patients at early stages of the disease such as after surgical removal of HCC and hepatic metastases of CRC, two diseases with increased likelihood of recurrence. Finally, new ways of long-term local delivery of signals inducing CD4+ T cell differentiation towards the Th1 lineage or vaccination against liver tumor antigens would eventually overcome the drawbacks of the pro-tolerogenic liver influence and the impairment or reduced immune response capacity caused by HBV/HCV viruses.

## Competing interests

The authors declare that they have no competing interests.

## Authors' contributions

MP and VR: The immune system and the induction of antitumor immunity – basic concepts; Contribution of adoptive T-cell therapy strategies. JBA: The liver: an immunological privileged organ. OGS and JBA: Immunostimulating monoclonal antibodies. LA and MM: Immunotherapy with dendritic cells. GM: Introduction; The liver: an immunological privileged organ; Systemic use of immunostimulatory cytokines; Gene transfer of cytokines and costimulatory molecules; Genetic vaccination; Conclusions.

MG did a deep revision of the English grammar and style because English is not our native language. CA constructed the figure and the tables. MS was involved in the analysis and revision of the data included in the paper. GM outlined the topics of the manuscript and invited to each author to write specific chapters of the paper. All authors read and approved the final manuscript.
